# Supplementary material for: Early circulating tumor DNA dynamics and clonal selection with palbociclib and fulvestrant for breast cancer
Source: Nat Commun. 2018 Mar 1;9:896. doi: 10.1038/s41467-018-03215-x (PMC5832789; doi:10.1038/s41467-018-03215-x)
Supplement: Supplementary file 1 — Supplementary Information [file 41467_2018_3215_MOESM1_ESM.pdf]

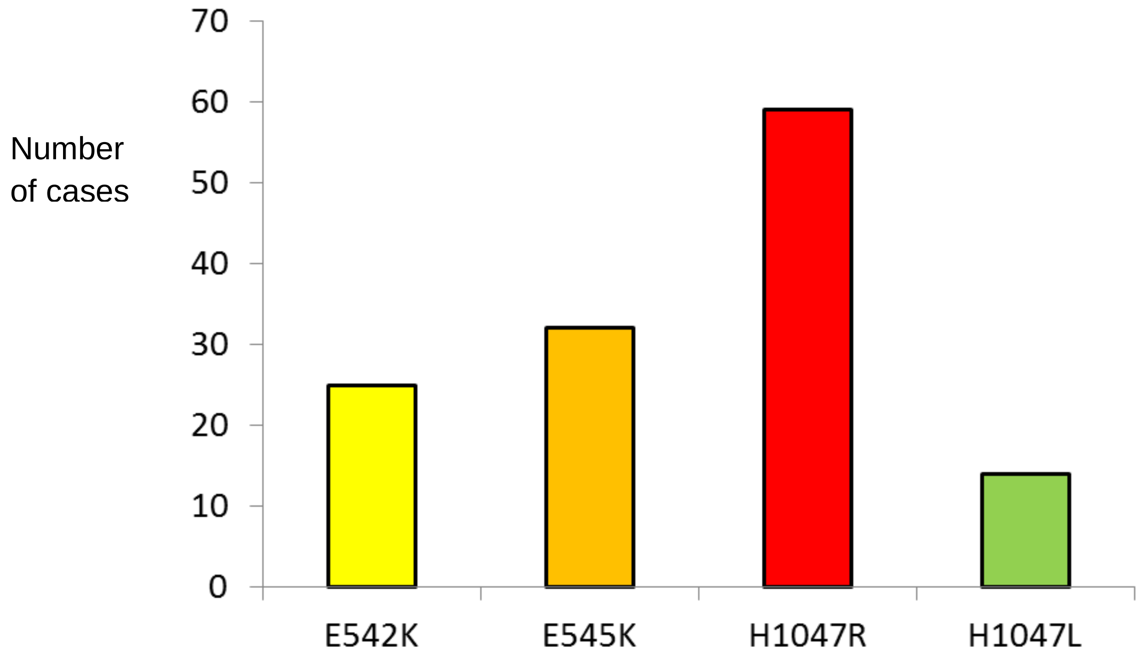

Supplementary figure 1. *PIK3CA* mutations identified in the baseline plasma samples of the PALOMA-3 trial with multiplex digital PCR and confirmed in singleplex.

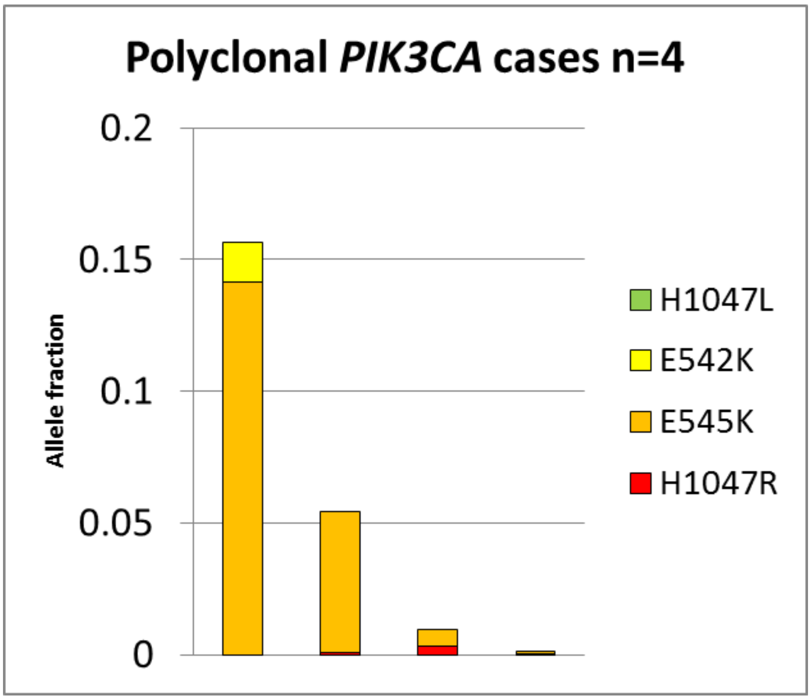

Sample	E545K	H1047R	E542K
52	0.0009	0.0005	0.0000
312	0.0077	0.0031	0.0000
34	0.1414	0.0000	0.0150
157	0.0538	0.0006	0.0000

Supplementary figure 2. Allele fraction details for polyclonal *PIK3CA* mutations observed in the PALOMA-3 set.

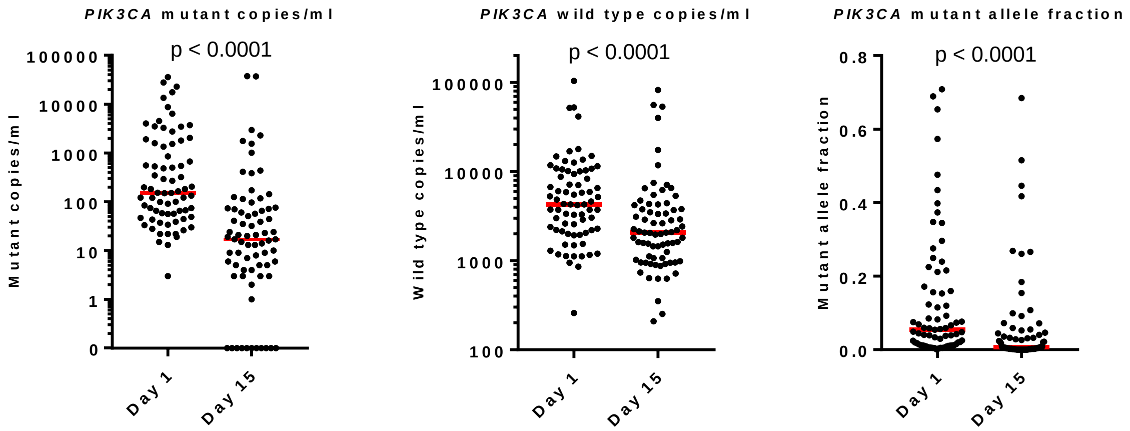

Supplementary figure 3. *PIK3CA* allele characteristics in the baseline plasma samples compared to day 15. P values Wilcoxon signed-rank test. Red line at median.

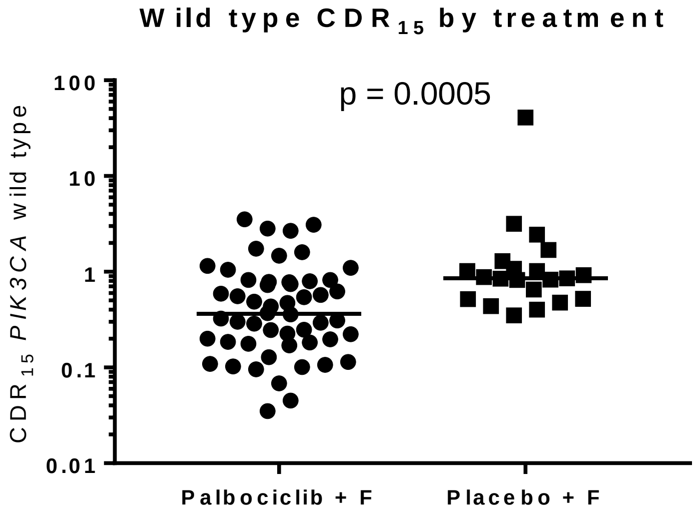

Supplementary figure 4. CDR_{15} for the wild type *PIK3CA* allele in patients with *PIK3CA* mutations split by treatment. CDR_{15} is defined as the ratio of mutant copies/ml at day 15 to the mutant copies/ml at day 1. F is fulvestrant. P value by Mann Whitney. Line at median. Correlation analyses of the absolute reduction of mutant and wild type copies/ml (*ESR1* Spearman's r = 0.57, 95%CI 0.38 – 0.72) p<0.0001 *PIK3CA* Spearman's r = 0.58 (95%CI 0.40 – 0.72, p<0.0001) suggest at least in part this is due to a reduction in detection of wild type allele from the tumour.

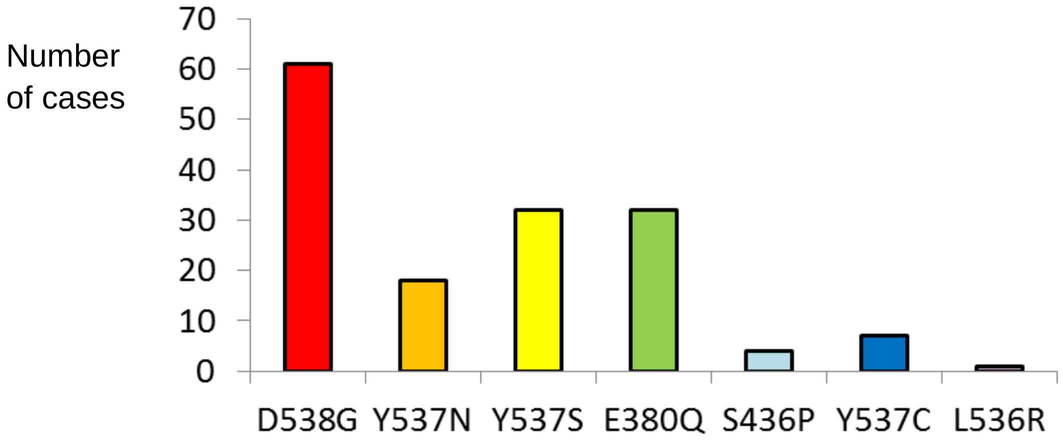

Supplementary figure 5. *ESR1* mutations identified in the baseline plasma samples of the PALOMA-3 trial with multiplex digital PCR and confirmed in singleplex.

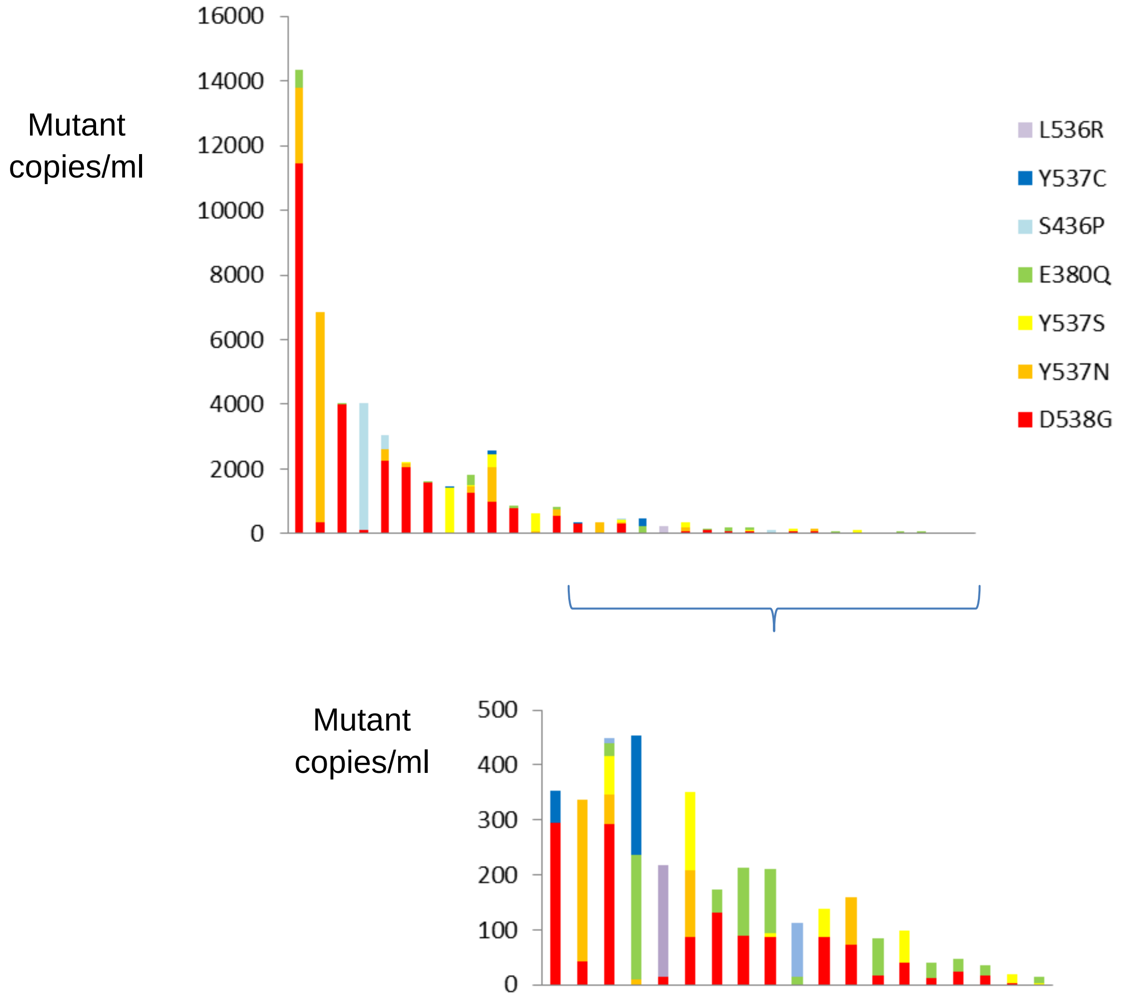

Supplementary figure 6. Clonal composition of polyclonal *ESR1* mutant samples at baseline by digital PCR. Each bar represents a single patient identified as having more than one *ESR1* mutation. The mutations are color-coded to demonstrate their proportion of contribution to the total mutant copies/ml. The lower panel is an enlarged section of the upper panel, to enable clearer visualisation of the lower abundance sub clones.

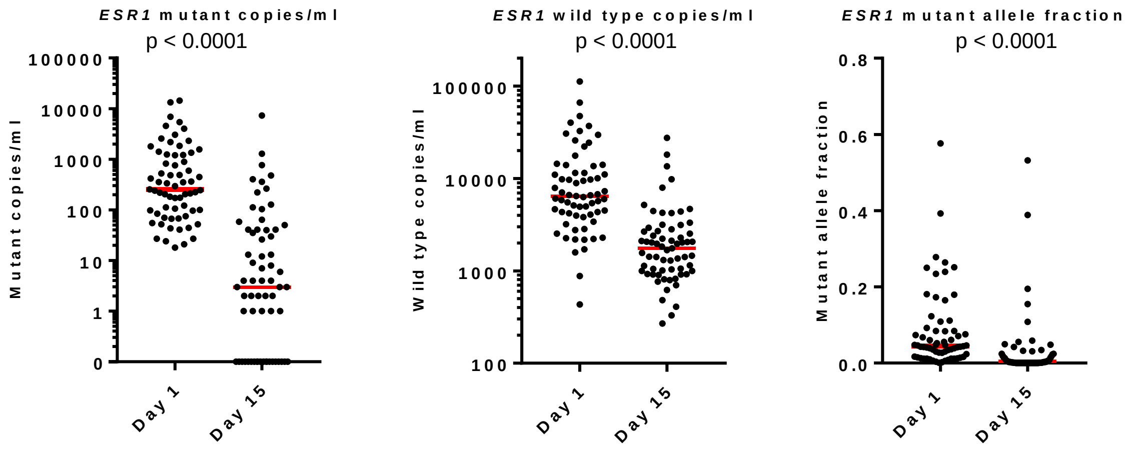

Supplementary figure 7. *ESR1* allele characteristics in the baseline plasma samples compared to day 15. P values Wilcoxon signed-rank test. Lines at median.

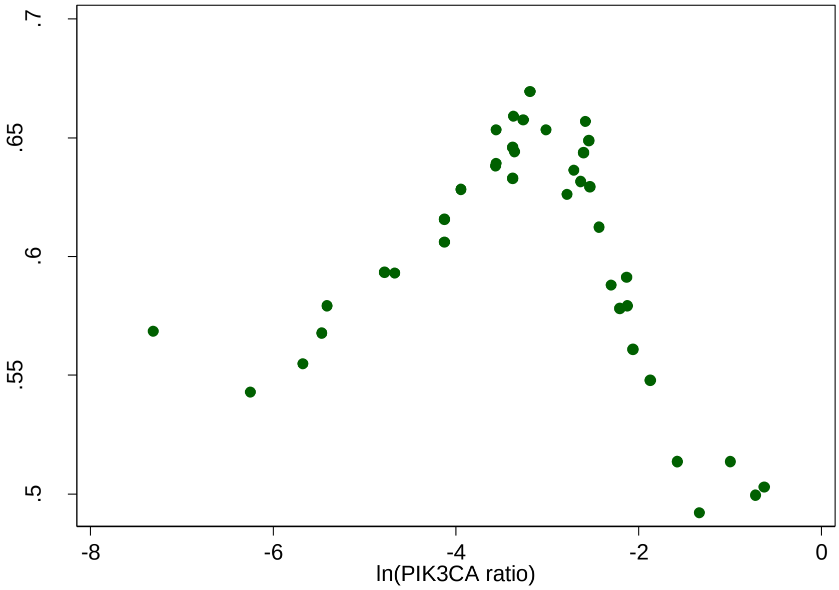

Cut-point	ln(Cut-point)	N ≤ cut-point	N > cut-point	HR (high cf. low)	Lower 95% CI	Upper 95% CI	Logrank p-value	Harrell's C	q-value*
0.0757347	-2.580519	36	16	4.75	2.03	11.12	0.0000893	0.657	0.0037
0.041325	-3.186287	30	22	4.92	1.98	12.26	0.0001756	0.669	0.007
0.0783447	-2.546637	37	15	4.4	1.89	10.2	0.000185	0.649	0.0072
0.0382593	-3.263368	29	23	4.25	1.8	10.07	0.0003809	0.657	0.0145
0.0345713	-3.364732	27	25	4.42	1.8	10.81	0.0004329	0.659	0.016
0.0741846	-2.601199	35	17	3.96	1.71	9.19	0.0005667	0.644	0.0204
0.0491763	-3.012343	31	21	4.18	1.73	10.06	0.000584	0.653	0.0204
0.0794812	-2.532235	38	14	3.82	1.65	8.82	0.000793	0.629	0.027
0.028587	-3.554804	24	28	4.3	1.68	11.03	0.0010564	0.653	0.0349
0.066741	-2.706935	33	19	3.77	1.61	8.86	0.0011213	0.636	0.0359

Supplementary figure 8. Optimizing circulating DNA response cut point by Harrell's c-index. Ln(*PIK3CA* ratio) is the natural logarithm of the CDR_{15} for *PIK3CA* mutation where the CDR_{15} is defined as the ratio of mutant copies/ml at day 15 and day 1.

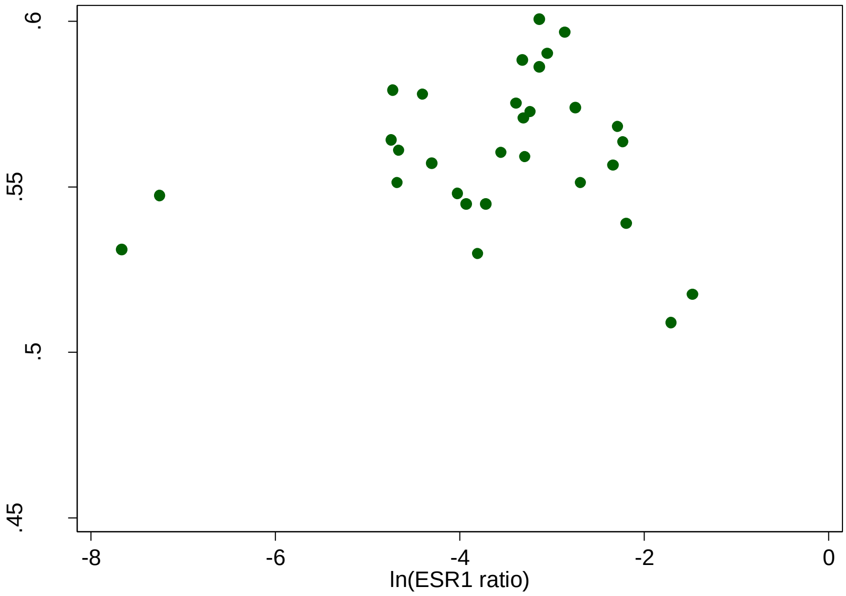

Cut-point	ln(Cut-point)	N ≤ cut-point	N > cut-point	HR (high cf. low)	Lower 95% CI	Upper 95% CI	Logrank p-value	Harrell's C	q-value*
0.043496	-3.13508	36	11	3.3	1.36	8.01	0.004989	0.601	0.1497
0.057136	-2.86232	38	9	3.65	1.35	9.85	0.006087	0.597	0.1765
0.047312	-3.05099	37	10	3.08	1.24	7.67	0.010586	0.59	0.2858
0.036092	-3.32169	31	16	2.33	1	5.41	0.042326	0.588	0.9156
0.043318	-3.13919	35	12	2.59	1.08	6.22	0.02603	0.586	0.6768
0.008871	-4.72496	20	27	1.77	0.79	3.97	0.157281	0.579	0.9156
0.012293	-4.39871	23	24	1.95	0.85	4.48	0.108305	0.578	0.9156
0.033794	-3.38748	30	17	2.04	0.88	4.75	0.088481	0.575	0.9156
0.064382	-2.74293	39	8	3	1.06	8.49	0.028348	0.574	0.7087
0.039353	-3.23519	34	13	2.16	0.9	5.16	0.074636	0.573	0.9156

Supplementary figure 9. Optimizing circulating DNA response cut point by Harrell's c-index for *ESR1* mutations. Ln(*ESR1* ratio) is the natural logarithm of the CDR_{15} for *ESR1* mutation where the CDR_{15} is defined as the ratio of mutant copies/ml at day 15 and day 1.

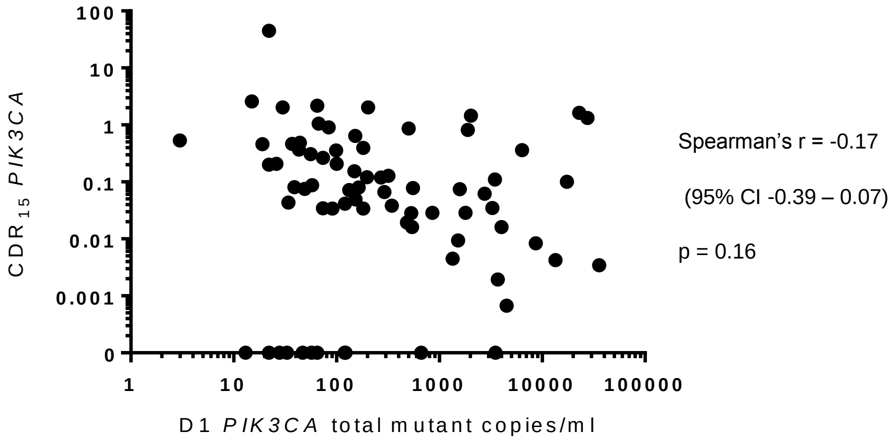

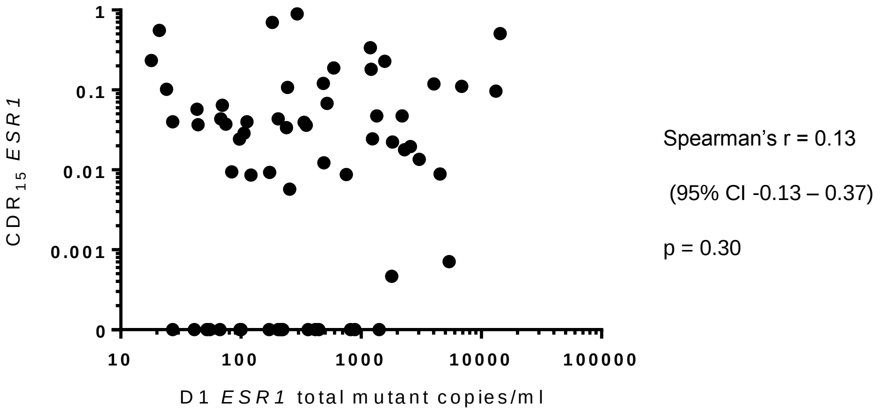

Supplementary figure 10. (Upper panel) Plot of *PIK3CA* day 1 mutant copies/ml versus *PIK3CA* CDR_{15}, Spearman's r, 2-sided p value (Lower panel) Plot of *ESR1* day 1 mutant copies/ml versus *ESR1* CDR_{15}, Spearman's r, 2-sided p value. HR – hazard ratio. CI – confidence interval.

Univariate analysis	Hazard ratio	Lower 95% CI	Upper 95% CI	p value
Prior hormonal therapy (Yes/No)	0.778	0.288	2.1	0.62
Baseline copies/ml mutant *PIK3CA*	1.22	0.606	2.43	0.582
Site of metastatic disease (Visceral/Non-visceral)	1.69	0.763	3.73	0.191
Menopausal status (Pre/peri v Post)	0.572	0.225	1.46	0.236
Number of prior therapies (1 v >1)	3.83	0.9	16.3	0.0502
Number of disease sites (1 v >1)	2.68	1.11	6.48	**0.0234**
Disease site liver (Yes v No)	3.39	1.5	7.64	**0.00181**
CDR15 (High v Low)	4.92	1.98	12.3	**0.000178**

Multivariate analysis	Hazard ratio	Lower 95% CI	Upper 95% CI	p value
Disease site liver (Yes v No)	4.01	1.76	9.15	**0.00095**
CDR15 (High v Low)	5.73	2.26	14.51	**0.00023**

Supplementary figure 11. Univariate and multivariate analyses of n=52 patients used to assess mutant *PIK3CA* CDR_{15} in the patients receiving palbociclib and fulvestrant. The multivariate analysis was performed using the Cox proportional hazards model. The baseline clinical or pathological factors included in the analysis were disease site (Visceral vs. Non-Visceral), sensitivity to prior hormonal therapy (Yes vs. No), menopausal status at study entry (Pre/Peri vs. Post), number of prior

therapies (1 vs. 2 or more), number of disease sites (1 vs. 2 or more), disease site (liver vs. other), and baseline copies of *PIK3CA*. Univariate analyses were conducted as an initial procedure to only retain factors with $p<0.1$ in the multivariate analysis. For the multivariate analysis predictors with $p<0.1$ from the univariate analyses were retained (4 out of 7). Two of the predictors stayed in the final model (met the typical $p<0.05$ criteria) with the other two predictors dropped (with p-value greater than 0.05 when all 4 predictors were included in the multivariable analysis). The assumption for Cox regression met and there was not interaction between the two variables.

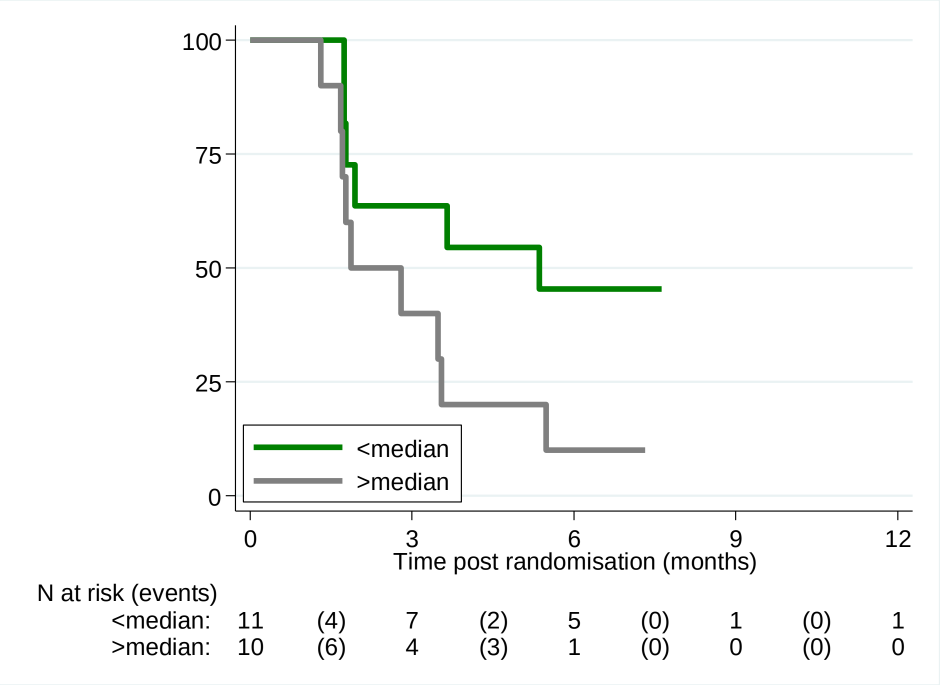

Supplementary figure 12. Kaplan Meier plot for PFS of patients randomized to placebo and fulvestrant split by median *PIK3CA* CDR_{15}. Hazard ratio for >median compared with <median = 2.54 (95% CI 0.89 – 7.25). Logrank test p=0.07.

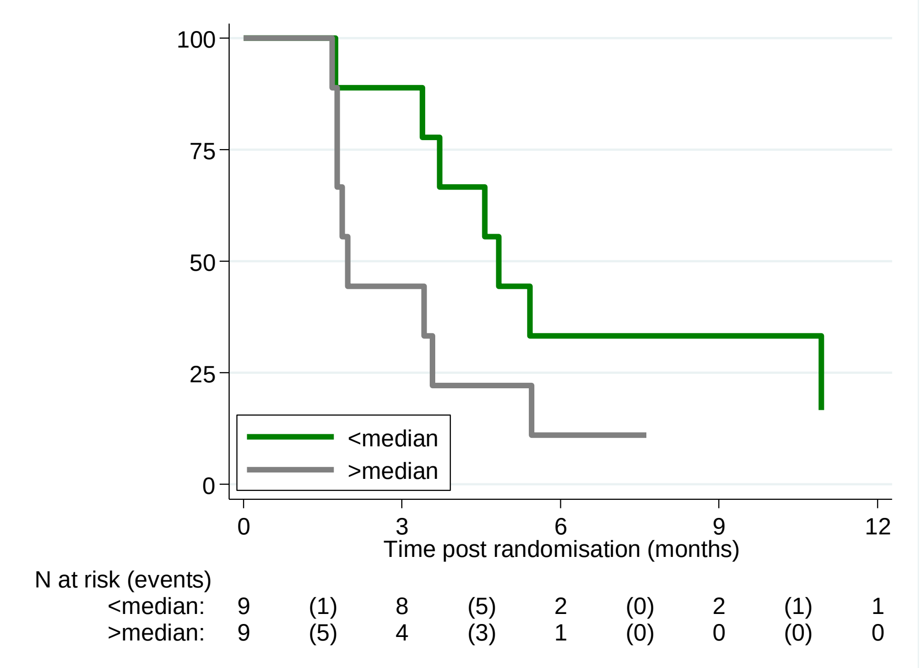

Supplementary figure 13. Kaplan Meier plot for PFS of patients randomized to placebo and fulvestrant split by median *ESR1* CDR_{15}. Hazard ratio for >median compared with <median = 2.28 (95% CI 0.77 – 6.70). Logrank test p=0.12.

Lab ID	D1 *PIK3CA* total mutant copies/ml	D15 *PIK3CA* total mutant copies/ml	D15/D1 *PIK3CA* total mutant copies/ml	D1 *ESR1* total mutant copies/ml	D15 *ESR1* total mutant copies/ml	D15/D1 *ESR1* total mutant copies/ml	Inferred *ESR1* mutant copies/ml	Below LOD
253	3708	7	0.002	886	0	0	2	TRUE
152	2751	171	0.062	415	0	0	26	FALSE
132	1584	117	0.074	27	0	0	2	TRUE
241	555	44	0.078	67	0	0	5	FALSE
72	542	9	0.016	212	0	0	3	TRUE
27	152	97	0.643	1414	0	0	909	FALSE
323	121	5	0.041	52	0	0	2	TRUE
85	58	5	0.088	55	0	0	5	FALSE

Supplementary figure 14. Inferred *ESR1* mutant copies/ml in cases with both *PIK3CA* and *ESR1* mutations and discordant *PIK3CA*/*ESR1* CDR_{15}. *ESR1* copies/ml inferred using linear extrapolation

from the *PIK3CA* CDR_{15} data then compared with the theoretical limit of detection for the assay. The theoretical limit of detection for a singleplex digital PCR assay was calculated on the basis of one FAM positive droplet out of 20,000 using the equation: Concentration = -ln(Negative droplets/All droplets)/Droplet volume * 20,000. It was then scaled by the tested plasma equivalent volume for inferred mutant copies/ml. Droplet volume was taken as 0.89nl.

Lab ID	Inferred *ESR1* mutant copies/ml	New LOD with extra volume analysed	D538G copies/ml	E380Q copies/ml	D15 *ESR1* total mutant copies/ml	D15/D1 *ESR1* total mutant copies/ml	Change in status
253	2	2	0	0	0	0	FALSE
132	2	2	N/A	0	0	0	FALSE
72	3	2	5	0	5	0.024	TRUE
323	2	2	0	N/A	0	0	FALSE

Supplementary figure 15. Results from testing further 0.25ml plasma equivalent for each *ESR1* mutation in thecases detailed in supplementary figure C with an inferred mutant/copies/ml below the theoretical limit of detection of the digital PCR assay.

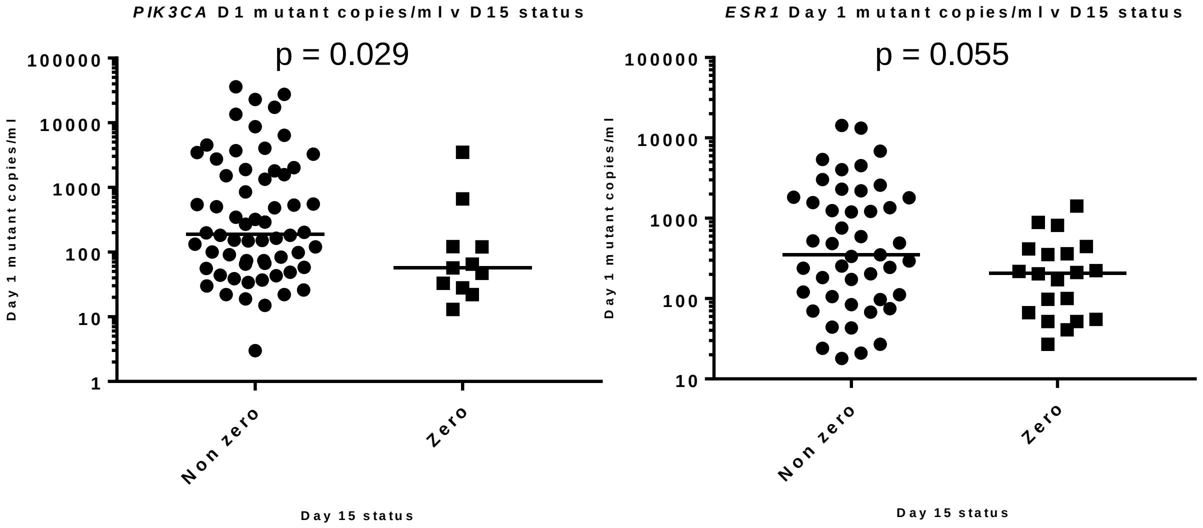

Supplementary figure 16. Comparison of baseline mutant copies/ml with detectable status at day 15 for *PIK3CA* and *ESR1*. P value calculated using Mann Whitney.

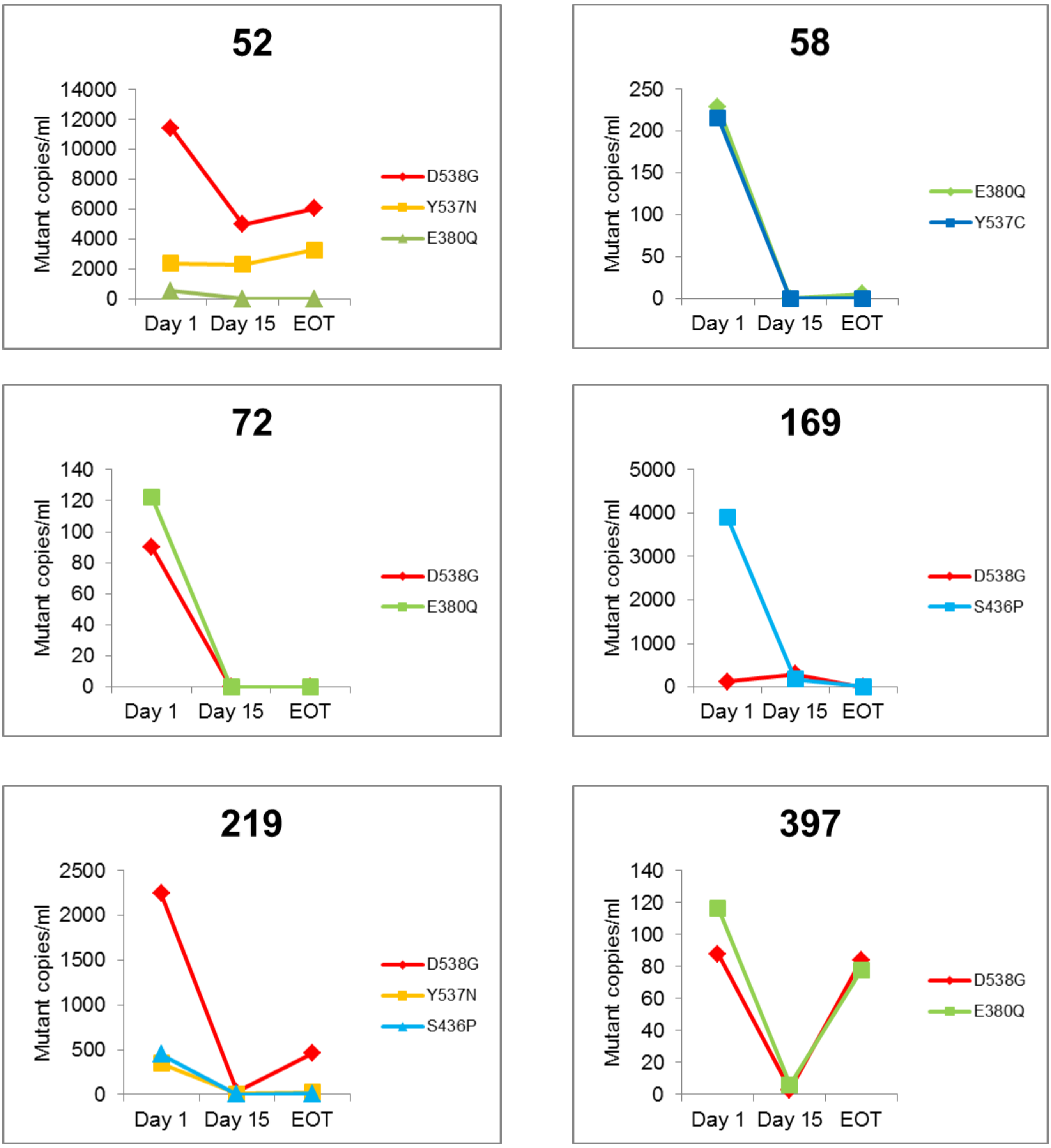

Supplementary figure 17. Clonal dynamics of polyclonal *ESR1* mutations. Data from 6 patients with polyclonal *ESR1* mutations identified at baseline then tracked through day 15 to end of treatment. Vertical axis in each plot is mutant copies/ml

Number of negative samples out of 25 in single simulation	2	3	4	5	6	7	8	9	10	11
Number of times outcome observed after 10,000 repeats	0	474	1887	2944	2642	1450	484	109	10	0

Supplementary table 1. Simulated data to assess the likelihood of sampling error explaining the number of discordant ESR1 undetectable tests in day 15 samples assessed for both *ESR1* and *PIK3CA* CDR_{15} (Figure 4D). To model sampling error we defined the probability of having a positive result for each day15 sample by using the inferred copies/ml of *ESR1*, assuming equivalent CDR_{15} to *PIK3CA* and independence of sampling events. This probability of *ESR1* being undetectable by sampling error was modelled using an individual Poisson distribution Po(λ) for each sample, where λ = inferred concentration and the probability of a negative result arising as a result of sampling error Po(X=0) = e-λ. The observed rate of undetectable *ESR1* was 10/25 samples (40%, Figure 4D). In the simulation, repeated 10,000 times, the frequency of 10 or more negatives was 0.1% (10/10,000).
